# P-2111. Automated Bacterial Growth Detection, Bacterial Species Estimation and Antimicrobial Susceptibility Testing on Agar Plates Using a Light Transmission-based Monitoring System

**DOI:** 10.1093/ofid/ofae631.2267

**Published:** 2025-01-29

**Authors:** Reiichi Ariizumi, Kyohei Umebayashi, Shogo Maeta, Hiroaki Ozaki, Akihiko Fujisawa, Tomoya Tezen, Takanori Tsunashima, Kaoru Ito, Daichi Abe, Kazunori Yamaguchi, Kenichiro Ohnuma, Nami Ishida, Kei Furui Ebisawa, Goh Ohji, Masakazu Nakajima, Makoto Taketani

**Affiliations:** CarbGeM Inc., Shibuya-ku, Tokyo, Japan; CarbGeM Inc., Shibuya-ku, Tokyo, Japan; CarbGeM Inc., Shibuya-ku, Tokyo, Japan; CarbGeM Inc., Shibuya-ku, Tokyo, Japan; Japan Display Inc., Ebina-shi, Kanagawa, Japan; Japan Display Inc., Ebina-shi, Kanagawa, Japan; Japan Display Inc., Ebina-shi, Kanagawa, Japan; Japan Display Inc., Ebina-shi, Kanagawa, Japan; Japan Display Inc., Ebina-shi, Kanagawa, Japan; Japan Display Inc., Ebina-shi, Kanagawa, Japan; Kobe University Hospital, Kobe, Hyogo, Japan; Kobe University Hospital, Kobe, Hyogo, Japan; Kobe University Hospital, Kobe, Hyogo, Japan; Kobe University Hospital, Kobe, Hyogo, Japan; CarbGeM Inc., Shibuya-ku, Tokyo, Japan; CarbGeM Inc., Shibuya-ku, Tokyo, Japan

## Abstract

**Background:**

Culturing bacteria on agar plates is essential for diagnosing bacterial infections, but it usually takes overnight and is labor-intensive. We have developed a novel device using a thin-film transistor image sensor to measure light transmission through agar plates every five minutes during incubation (Nakada et al. ASM 2023 and IDweek 2023). Here we present a proof of concept study for using this device for automated early detection and disk-diffusion susceptibility testing and compare the results to those obtained by experienced microbiology technicians.

**Figure:**
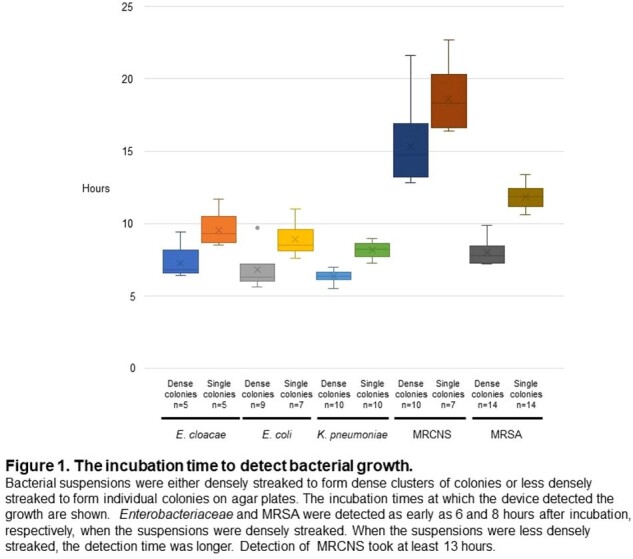

**Methods:**

Chromogenic agar plates were used for early detection and subsequent species estimation based on colony color changes, i.e. *Enterobacteriaceae* on CHROMagar mSuper CARBA plates and *Staphylococci* on MDRS-K plates. Statistical background subtraction was used for early detection of bacterial growth and histogram comparison of pseudo-color images created by correcting the intensity of red, blue, and green lights for species estimation.

For disk diffusion tests susceptible and resistant *E. coli* and *S. aureus* were inoculated on Mueller-Hinton plates against the known agents and incubated for 18 hours with monitoring. Transmitted light images after 18 hours were first binarized and the distance of the shortest bacterial growth pixel from the center of the corresponding antibiotic disk was measured as the inhibition zone diameter.

**Figure:**
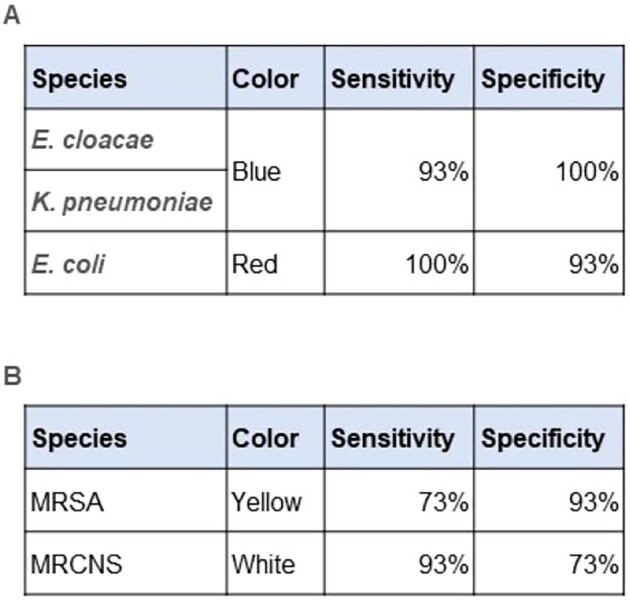

**Results:**

The device detected *Enterobacteriaceae* and MRSA as early as 6 and 8 hours after incubation, respectively, when the suspensions were densely streaked (Figure 1).

The device also estimated the bacterial species with sensitivity and specificity over 90% for Enterobacteriaceae, however, the specificity for MRSA against MRCNS was approximately 70% (Table 1).

An example of the automated inhibition zone measurement is shown in Figure 2.

All automated measurements agreed well with the manual measurements (Table 2).

**Figure:**
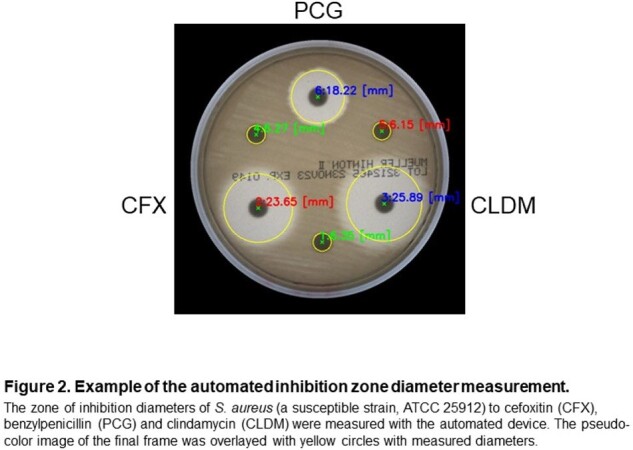

**Conclusion:**

The performance in the bacterial detection, species estimation and the zone diameter measurement indicated that the device can be useful in accelerating, automating, and standardizing routine testing in microbiology laboratories.

**Figure:**
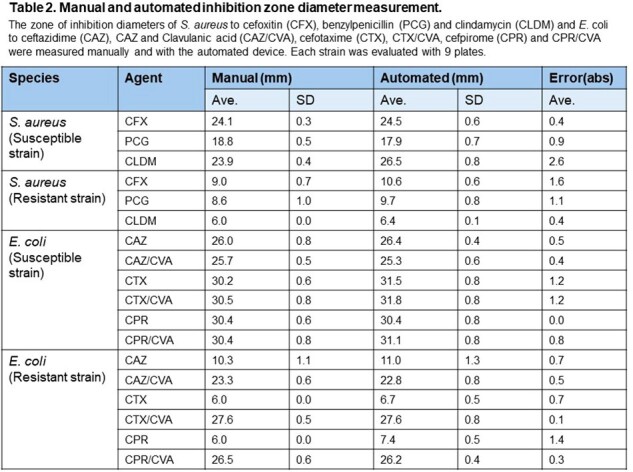

**Disclosures:**

Goh Ohji, MD, Ph.D, CarbGem Inc.: Advisor/Consultant Masakazu Nakajima, B. Engineering, CarbGeM Inc.: Board Member|CarbGeM Inc.: Ownership Interest

